# Supplementary material for: Genome-Wide Characterization of Soybean 1-Aminocyclopropane-1-carboxylic Acid Synthase Genes Demonstrates the Importance of GmACS15 in the Salt Stress Responses
Source: Int J Mol Sci. 2025 Mar 12;26(6):2526. doi: 10.3390/ijms26062526 (PMC11942397; doi:10.3390/ijms26062526)
Supplement: Supplementary file 1 [file ijms-26-02526-s001.zip › ijms-3451899-supplementary.pdf]

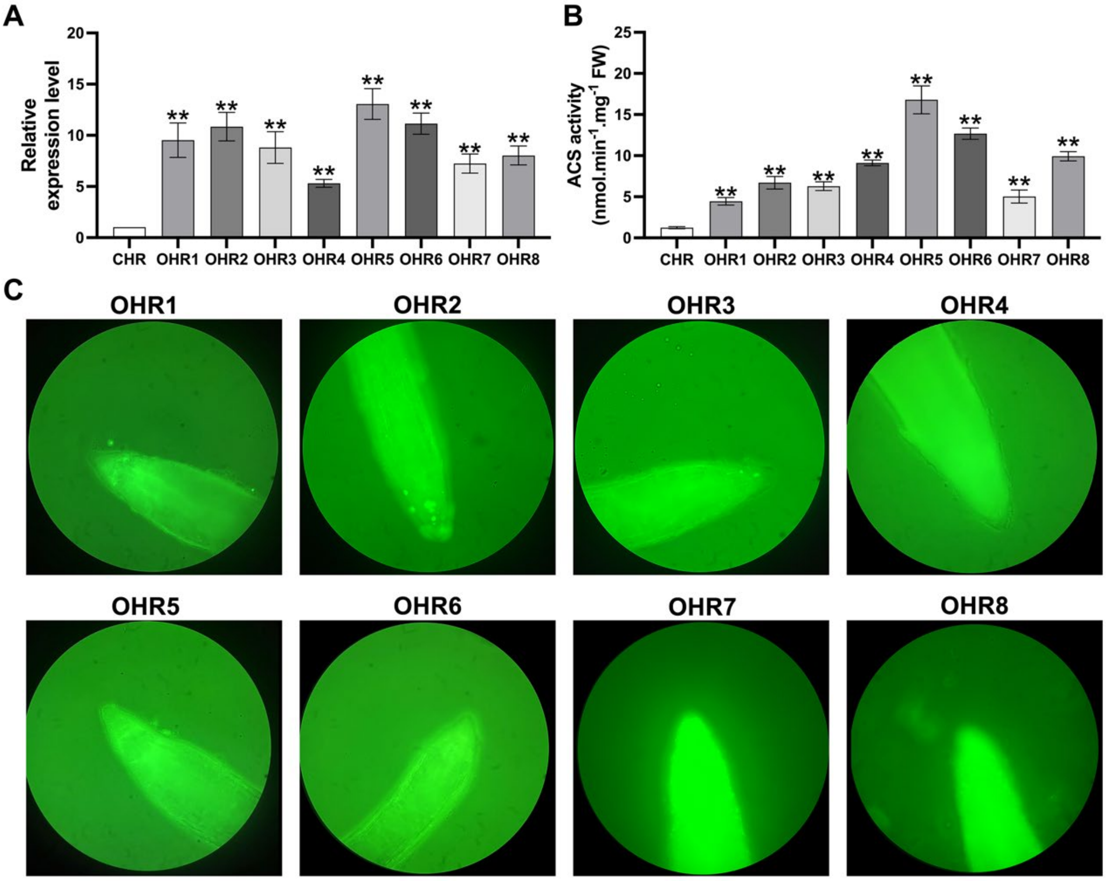


**Figure S1.** Identification of transgene expression in *GmACS15* transgenic soybean hairy roots. **(A)** The expression levels and **(B)** enzyme activities of *GmACS15* in *GmACS15*-overexpressing hairy roots compared with the control hairy roots (WT). **(C)** Pictures were taken approximately 2 weeks after transformation to check for GFP fluorescence. Bars=100 μm.

**Table S1.** The gene ID and information of *ACS* used in this study.

| Gene Name | Gene ID | Gene Location | | |
|---|---|---|---|---|
| | | Chromosome | Start | End |
| GmACS1 | Glyma.01G003900 | Chr1 | 411125 | 413137 |
| GmACS2 | Glyma.01G196100 | Chr1 | 53027373 | 53030242 |
| GmACS3 | Glyma.01G213700 | Chr1 | 54486994 | 54489957 |
| GmACS4 | Glyma.03G025100 | Chr3 | 2719351 | 2719869 |
| GmACS5 | Glyma.04G048700 | Chr4 | 3941607 | 3943357 |
| GmACS6 | Glyma.05G108900 | Chr5 | 28858965 | 28861063 |
| GmACS7 | Glyma.05G211700 | Chr5 | 39315581 | 39318444 |
| GmACS8 | Glyma.05G223000 | Chr5 | 40248224 | 40250093 |
| GmACS9 | Glyma.06G049900 | Chr6 | 1526712 | 1529646 |
| GmACS10 | Glyma.07G065700 | Chr7 | 5923612 | 5927198 |
| GmACS11 | Glyma.07G128000 | Chr7 | 15313240 | 15315484 |
| GmACS12 | Glyma.08G018000 | Chr8 | 1450278 | 1453241 |
| GmACS13 | Glyma.08G030100 | Chr8 | 2411044 | 2413124 |
| GmACS14 | Glyma.09G152700 | Chr9 | 37521670 | 37525557 |
| GmACS15 | Glyma.09G255000 | Chr9 | 47428922 | 47432420 |
| GmACS16 | Glyma.11G021500 | Chr11 | 1526712 | 1529646 |
| GmACS17 | Glyma.11G028200 | Chr11 | 2023320 | 2026136 |
| GmACS18 | Glyma.11G045600 | Chr11 | 3379030 | 3381262 |
| GmACS19 | Glyma.16G032200 | Chr16 | 3050719 | 3054820 |
| GmACS20 | Glyma.16G203600 | Chr16 | 36457598 | 36461359 |
| GmACS21 | Glyma.17G158100 | Chr17 | 13554133 | 13556242 |
| GmACS22 | Glyma.18G238100 | Chr18 | 52690346 | 52694060 |
| OsACS1 | LOC_Os01g09700 | Chr1 | 5002117 | 5005727 |
| OsACS2 | LOC_Os03g51740 | Chr3 | 29660661 | 29662927 |
| OsACS3 | LOC_Os04g48850 | Chr4 | 29131809 | 29134682 |
| OsACS4 | LOC_Os05g10780 | Chr5 | 5954550 | 5956246 |
| OsACS5 | LOC_Os05g25490 | Chr5 | 14821903 | 14824477 |
| OsACS6 | LOC_Os06g03990 | Chr6 | 1629716 | 1633629 |
| AtACS1 | AT1G01480 | Chr1 | 175781 | 178400 |
| AtACS2 | AT2G22810 | Chr2 | 9717790 | 9719472 |
| AtACS3 | AT3G49700 | Chr3 | 18434469 | 18436141 |
| AtACS4 | AT3G61510 | Chr3 | 22763494 | 22765730 |
| AtACS5 | AT4G08040 | Chr4 | 4887111 | 4888939 |
| AtACS6 | AT4G11280 | Chr4 | 6864096 | 6866116 |
| AtACS7 | AT4G26200 | Chr4 | 13275306 | 13277188 |

| | | | | |
|---|---|---|---|---|
| AtACS8 | AT4G37770 | Chr4 | 17752083 | 17754157 |
| AtACS9 | AT5G65800 | Chr5 | 26330745 | 26332812 |
| MtACS1 | Medtr2g090730 | chr2 | 38881462 | 38884174 |
| MtACS2 | Medtr3g103550 | chr3 | 47826473 | 47828998 |
| MtACS3 | Medtr4g097540 | chr4 | 40206877 | 40208967 |
| MtACS4 | Medtr5g011400 | chr5 | 3262516 | 3265496 |
| MtACS5 | Medtr5g015020 | chr5 | 5133747 | 5135930 |
| MtACS6 | Medtr6g091760 | chr6 | 34566027 | 34568470 |
| MtACS7 | Medtr7g079080 | chr7 | 29963155 | 29966691 |
| MtACS8 | Medtr8g098930 | chr8 | 41419292 | 41422107 |
| MtACS9 | Medtr8g101750 | chr8 | 42788673 | 42790665 |
| MtACS10 | Medtr8g028600 | chr8 | 10966038 | 10969861 |
| MtACS11 | Medtr8g021970 | chr8 | 7754455 | 7755976 |
| MtACS12 | Medtr8g101820 | chr8 | 42798281 | 42800233 |
| TaACS1 | TraesCS2A02G396400.1 | chr2A | 650243368 | 650246266 |
| TaACS2 | TraesCS2B02G414800.1 | chr2B | 592801835 | 592804641 |
| TaACS3 | TraesCS2D02G394200.1 | chr2D | 505814183 | 505817055 |
| TaACS4 | TraesCS3A02G106600.1 | chr3A | 70848330 | 70850223 |
| TaACS5 | TraesCS3B02G125200.1 | chr3B | 99500619 | 99502567 |
| TaACS6 | TraesCS3D02G108700.1 | chr3D | 61802605 | 61804484 |
| TaACS7 | TraesCS4A02G256500.1 | chr4A | 568192758 | 568195091 |
| TaACS8 | TraesCS4A02G422900.1 | chr4A | 693274378 | 693278701 |
| TaACS9 | TraesCS4B02G058200.1 | chr4B | 48991437 | 48993796 |
| TaACS10 | TraesCS4D02G058200.1 | chr4D | 34088249 | 34091236 |
| TaACS11 | TraesCS7A02G066400.1 | chr7A | 33361446 | 33365622 |
| TaACS12 | TraesCS7D02G060600.1 | chr7D | 33045597 | 33051044 |

**Table S2.** The primers used in this study.

| Primer Names | Sequence (5'-3') | Description |
|---|---|---|
| GmACS1-F | ATGGGTATTGAGATGGAGCAACC | cloning |
| GmACS1-R | AAAAAAGACGTTTTGTGTCCTTATTC | cloning |
| GmACS8-F | ATGGGTATCAAGATTGAGCAAGAGC | cloning |
| GmACS8-R | CTCTTTCTTCATTCGTTCCATGAA | cloning |
| GmACS15-F | ATGGCTTTGGGGAACAACAGT | cloning |
| GmACS15-R | AGTGGCTCTGACAAGGGGTGA | cloning |
| pGEX-4T-3-GmACS15-F | ATGGCTTTGGGGAACAACAGTCA | cloning |
| pGEX-4T-3-GmACS15-R | AGTGGCTCTGACAAGG | cloning |
| GmACS1-qF | GTTCTTCACAAGCGTGGCAG | qRT-PCR |
| GmACS1-qR | TGGTTCCTCTTCCTCAGCCT | qRT-PCR |
| GmACS2-qF | TTTTGCCTCGCGGAAAAAGG | qRT-PCR |
| GmACS2-qR | GTAGGTTCAGTTCACCCCGG | qRT-PCR |
| GmACS3-qF | GGTTGAGGCTGCGGAAAATG | qRT-PCR |
| GmACS3-qR | CAAGAGGAACCCGGGGTAAC | qRT-PCR |
| GmACS4-qF | GCACTGCTGAAGGAGTAAACC | qRT-PCR |
| GmACS4-qR | CAGCATCAGCCAAACAGAACA | qRT-PCR |
| GmACS5-qF | CGGTCTTCGACTCACCCAAA | qRT-PCR |
| GmACS5-qR | TGAGGACACCAATCCGAAGC | qRT-PCR |
| GmACS6-qF | AACACGTTCAAGGCGGAGAT | qRT-PCR |
| GmACS6-qR | AGAGCACCAGCACTACAACC | qRT-PCR |
| GmACS7-qF | TTTGTTTGGCAGACCCTGGT | qRT-PCR |
| GmACS7-qR | TGGTGATGAGCAAGCCCTTT | qRT-PCR |
| GmACS8-qF | AGGGTTGGAAAGTGTTGGCA | qRT-PCR |
| GmACS8-qR | CTCTCCAATGCCACCTCCAG | qRT-PCR |
| GmACS9-qF | AGATGCTTGTGTCTGGCCTC | qRT-PCR |
| GmACS9-qR | CGCCGCATTGCAACTTCTAA | qRT-PCR |
| GmACS10-qF | CGATTCCCAAGCAATGCTGG | qRT-PCR |
| GmACS10-qR | GCACGAATCCTGTTGAGTGC | qRT-PCR |
| GmACS11-qF | AGCAACCTTGTGTGGAGCTT | qRT-PCR |
| GmACS11-qR | TTTCCCCATGTGGAAGCCTC | qRT-PCR |
| GmACS12-qF | GATGGCTGCGAACCAAACTC | qRT-PCR |
| GmACS12-qR | GAGTGCAAATGGAGGCCTCT | qRT-PCR |
| GmACS13-qF | GGCAGAGGCTGAAGAAGAGG | qRT-PCR |

| | | |
|---|---|---|
| GmACS13-qR | CGTAGTCTCTCCAGTGCCAC | qRT-PCR |
| GmACS14-qF | ACGTCGATTTCCAAACCCGA | qRT-PCR |
| GmACS14-qR | TTCACGGATCCACCCATCAC | qRT-PCR |
| GmACS15-qF | ATTGTCCTGAGCCTGGTTGG | qRT-PCR |
| GmACS15-qR | AAGGGGTGAGTGAGGCATTG | qRT-PCR |
| GmACS16-qF | AAGAGGCGTACGAGAAAGGC | qRT-PCR |
| GmACS16-qR | ATGTGGATAAGGTCGCGGTC | qRT-PCR |
| GmACS17-qF | GCCACTTCTGCCATCGAGAT | qRT-PCR |
| GmACS17-qR | CCAGCAGGATTTGAGGGGTT | qRT-PCR |
| GmACS18-qF | CCTCATGTTTTGCCTCGCTG | qRT-PCR |
| GmACS18-qR | TTTGACTCTGAGGTTGCGCT | qRT-PCR |
| GmACS19-qF | ATGGGGTTTCCTGGCTTCAG | qRT-PCR |
| GmACS19-qR | AAAAGTCCCGCATTGCTTGG | qRT-PCR |
| GmACS20-qF | GGGTTTCGCGAGATCCCTAC | qRT-PCR |
| GmACS20-qR | CCCCCAATCACCTGATGCAT | qRT-PCR |
| GmACS21-qF | TTTTAGCTCCCCAGGCTTCG | qRT-PCR |
| GmACS21-qR | AGCATGGCAGAGAGGAGGTA | qRT-PCR |
| GmACS22-qF | TTCGTGGAGAGGTTTCTGGC | qRT-PCR |
| GmACS22-qR | CCCAGGTGACACATTGAGCT | qRT-PCR |
| GmACTIN4-qF | GTGTCAGCCATACTGTCCCCATTT | qRT-PCR |
| GmACTIN4-qR | GTTTCAAGCTCTTGCTCGTAATCA | qRT-PCR |

| **Table S3.** The syntenic relationships among ACSs | | | | | | | | | |
|---|---|---|---|---|---|---|---|---|---|
| ***Glycine max* gene location** | | | ***Glycine max* gene ID** | ***Glycine max* gene name** | ***Glycine max* gene location** | | | ***Glycine max* gene ID** | ***Glycine max* gene name** |
| **Chr** | **start** | **end** | | | **Chr** | **start** | **end** | | |
| Gm1 | 411125 | 413137 | Glyma.01G003900 | GmACS1 | Gm5 | 40248224 | 40250093 | Glyma.05G223000 | GmACS8 |
| Gm1 | 411125 | 413137 | Glyma.01G003900 | GmACS1 | Gm7 | 15313240 | 15315484 | Glyma.07G128000 | GmACS11 |
| Gm1 | 411125 | 413137 | Glyma.01G003900 | GmACS1 | Gm8 | 2411044 | 2413124 | Glyma.08G030100 | GmACS13 |
| Gm1 | 53027373 | 53030242 | Glyma.01G196100 | GmACS2 | Gm4 | 3941607 | 3943357 | Glyma.04G048700 | GmACS5 |
| Gm1 | 53027373 | 53030242 | Glyma.01G196100 | GmACS2 | Gm5 | 28858965 | 28861063 | Glyma.05G108900 | GmACS6 |
| Gm1 | 53027373 | 53030242 | Glyma.01G196100 | GmACS2 | Gm6 | 1526712 | 1529646 | Glyma.06G049900 | GmACS9 |
| Gm1 | 53027373 | 53030242 | Glyma.01G196100 | GmACS2 | Gm11 | 3379030 | 3381262 | Glyma.11G045600 | GmACS18 |
| Gm1 | 53027373 | 53030242 | Glyma.01G196100 | GmACS2 | Gm17 | 13554133 | 13556242 | Glyma.17G158100 | GmACS21 |
| Gm1 | 54486994 | 54489957 | Glyma.01G213700 | GmACS3 | Gm9 | 37521670 | 37525557 | Glyma.09G152700 | GmACS14 |
| Gm1 | 54486994 | 54489957 | Glyma.01G213700 | GmACS3 | Gm11 | 2023320 | 2026136 | Glyma.11G028200 | GmACS17 |
| Gm1 | 54486994 | 54489957 | Glyma.01G213700 | GmACS3 | Gm16 | 36457598 | 36461359 | Glyma.16G203600 | GmACS20 |
| Gm4 | 3941607 | 3943357 | Glyma.04G048700 | GmACS5 | Gm5 | 28858965 | 28861063 | Glyma.05G108900 | GmACS6 |
| Gm4 | 3941607 | 3943357 | Glyma.04G048700 | GmACS5 | Gm6 | 1526712 | 1529646 | Glyma.06G049900 | GmACS9 |

| | | | | | | | | | |
|---|---|---|---|---|---|---|---|---|---|
| Gm4 | 3941607 | 3943357 | Glyma.04G048700 | GmACS5 | Gm11 | 3379030 | 3381262 | Glyma.11G045600 | GmACS18 |
| Gm4 | 3941607 | 3943357 | Glyma.04G048700 | GmACS5 | Gm17 | 13554133 | 13556242 | Glyma.17G158100 | GmACS21 |
| Gm5 | 28858965 | 28861063 | Glyma.05G108900 | GmACS6 | Gm6 | 1526712 | 1529646 | Glyma.06G049900 | GmACS9 |
| Gm5 | 28858965 | 28861063 | Glyma.05G108900 | GmACS6 | Gm11 | 3379030 | 3381262 | Glyma.11G045600 | GmACS18 |
| Gm5 | 28858965 | 28861063 | Glyma.05G108900 | GmACS6 | Gm17 | 13554133 | 13556242 | Glyma.17G158100 | GmACS21 |
| Gm5 | 39315581 | 39318444 | Glyma.05G211700 | GmACS7 | Gm7 | 5923612 | 5927198 | Glyma.07G065700 | GmACS10 |
| Gm5 | 39315581 | 39318444 | Glyma.05G211700 | GmACS7 | Gm8 | 1450278 | 1453241 | Glyma.08G018000 | GmACS12 |
| Gm5 | 39315581 | 39318444 | Glyma.05G211700 | GmACS7 | Gm9 | 47428922 | 47432420 | Glyma.09G255000 | GmACS15 |
| Gm5 | 39315581 | 39318444 | Glyma.05G211700 | GmACS7 | Gm11 | 1526712 | 1529646 | Glyma.11G021500 | GmACS16 |
| Gm5 | 39315581 | 39318444 | Glyma.05G211700 | GmACS7 | Gm16 | 3050719 | 3054820 | Glyma.09G255000 | GmACS19 |
| Gm5 | 39315581 | 39318444 | Glyma.05G211700 | GmACS7 | Gm18 | 52690346 | 52694060 | Glyma.18G238100 | GmACS22 |
| Gm5 | 40248224 | 40250093 | Glyma.05G223000 | GmACS8 | Gm7 | 15313240 | 15315484 | Glyma.07G128000 | GmACS11 |
| Gm5 | 40248224 | 40250093 | Glyma.05G223000 | GmACS8 | Gm8 | 2411044 | 2413124 | Glyma.08G030100 | GmACS13 |
| Gm6 | 1526712 | 1529646 | Glyma.06G049900 | GmACS9 | Gm11 | 3379030 | 3381262 | Glyma.11G045600 | GmACS18 |
| Gm6 | 1526712 | 1529646 | Glyma.06G049900 | GmACS9 | Gm17 | 13554133 | 13556242 | Glyma.17G158100 | GmACS21 |
| Gm7 | 5923612 | 5927198 | Glyma.07G065700 | GmACS10 | Gm8 | 1450278 | 1453241 | Glyma.09G255000 | GmACS12 |
| Gm7 | 5923612 | 5927198 | Glyma.07G065700 | GmACS10 | Gm9 | 47428922 | 47432420 | Glyma.09G255000 | GmACS15 |

| | | | | | | | | | |
|---|---|---|---|---|---|---|---|---|---|
| Gm7 | 5923612 | 5927198 | Glyma.07G065700 | GmACS10 | Gm11 | 1526712 | 1529646 | Glyma.11G021500 | GmACS16 |
| Gm7 | 5923612 | 5927198 | Glyma.07G065700 | GmACS10 | Gm16 | 3050719 | 3054820 | Glyma.16G032200 | GmACS19 |
| Gm7 | 5923612 | 5927198 | Glyma.07G065700 | GmACS10 | Gm18 | 52690346 | 52694060 | Glyma.18G238100 | GmACS22 |
| Gm7 | 15313240 | 15315484 | Glyma.07G128000 | GmACS11 | Gm8 | 2411044 | 2413124 | Glyma.08G030100 | GmACS13 |
| Gm7 | 15313240 | 15315484 | Glyma.07G128000 | GmACS11 | Gm17 | 13554133 | 13556242 | Glyma.17G158100 | GmACS21 |
| Gm8 | 1450278 | 1453241 | Glyma.08G018000 | GmACS12 | Gm9 | 47428922 | 47432420 | Glyma.09G255000 | GmACS15 |
| Gm8 | 1450278 | 1453241 | Glyma.08G018000 | GmACS12 | Gm11 | 1526712 | 1529646 | Glyma.11G021500 | GmACS16 |
| Gm8 | 1450278 | 1453241 | Glyma.08G018000 | GmACS12 | Gm16 | 3050719 | 3054820 | Glyma.16G032200 | GmACS19 |
| Gm8 | 1450278 | 1453241 | Glyma.08G018000 | GmACS12 | Gm18 | 52690346 | 52694060 | Glyma.18G238100 | GmACS22 |
| Gm9 | 37521670 | 37525557 | Glyma.09G152700 | GmACS14 | Gm11 | 2023320 | 2026136 | Glyma.11G028200 | GmACS17 |
| Gm9 | 37521670 | 37525557 | Glyma.09G152700 | GmACS14 | Gm16 | 36457598 | 36461359 | Glyma.16G203600 | GmACS20 |
| Gm9 | 47428922 | 47432420 | Glyma.09G255000 | GmACS15 | Gm11 | 1526712 | 1529646 | Glyma.11G021500 | GmACS16 |
| Gm9 | 47428922 | 47432420 | Glyma.09G255000 | GmACS15 | Gm16 | 3050719 | 3054820 | Glyma.09G255000 | GmACS19 |
| Gm9 | 47428922 | 47432420 | Glyma.09G255000 | GmACS15 | Gm18 | 52690346 | 52694060 | Glyma.18G238100 | GmACS22 |
| Gm11 | 15313240 | 15315484 | Glyma.11G028200 | GmACS17 | Gm16 | 36457598 | 36461359 | Glyma.16G203600 | GmACS20 |
| Gm11 | 15313240 | 15315484 | Glyma.11G045600 | GmACS18 | Gm17 | 13554133 | 13556242 | Glyma.17G158100 | GmACS21 |
| Gm16 | 3050719 | 3054820 | Glyma.16G032200 | GmACS19 | Gm11 | 1526712 | 1529646 | Glyma.11G021500 | GmACS16 |

| | | | | | | | | | |
|---|---|---|---|---|---|---|---|---|---|
| Gm16 | 3050719 | 3054820 | Glyma.16G032200 | GmACS19 | Gm18 | 52690346 | 52694060 | Glyma.18G238100 | GmACS22 |
| Gm18 | 52690346 | 52694060 | Glyma.18G238100 | GmACS22 | Gm11 | 1526712 | 1529646 | Glyma.11G021500 | GmACS16 |
| ***Glycine max* gene location** | | | ***Glycine max* gene ID** | ***Glycine max* gene name** | ***Arabidopsis thaliana* gene location** | | | ***Zea mays* gene ID** | ***Zea mays* gene name** |
| **Chr** | **start** | **end** | | | **Chr** | **start** | **end** | | |
| Gm18 | 52690346 | 52694060 | Glyma.18G238100 | GmACS22 | At1 | 175781 | 178400 | AT1G01480 | AtACS1 |
| Gm16 | 3050719 | 3054820 | Glyma.16G032200 | GmACS19 | At1 | 175781 | 178400 | AT1G01480 | AtACS1 |
| Gm9 | 47428922 | 47432420 | Glyma.09G255000 | GmACS15 | At1 | 175781 | 178400 | AT1G01480 | AtACS1 |
| Gm7 | 5923612 | 5927198 | Glyma.07G065700 | GmACS10 | At1 | 175781 | 178400 | AT1G01480 | AtACS1 |
| Gm18 | 52690346 | 52694060 | Glyma.18G238100 | GmACS22 | At3 | 22763494 | 22765730 | AT3G61510 | AtACS4 |
| Gm16 | 3050719 | 3054820 | Glyma.16G032200 | GmACS19 | At3 | 22763494 | 22765730 | AT3G61510 | AtACS4 |
| Gm9 | 47428922 | 47432420 | Glyma.09G255000 | GmACS15 | At3 | 22763494 | 22765730 | AT3G61510 | AtACS4 |
| Gm7 | 5923612 | 5927198 | Glyma.07G065700 | GmACS10 | At3 | 22763494 | 22765730 | AT3G61510 | AtACS4 |
| Gm5 | 39315581 | 39318444 | Glyma.05G211700 | GmACS7 | At3 | 22763494 | 22765730 | AT3G61510 | AtACS4 |
| Gm5 | 39315581 | 39318444 | Glyma.05G211700 | GmACS7 | At4 | 6864096 | 6866116 | AT4G11280 | AtACS6 |
| ***Glycine max* gene location** | | | ***Glycine max* gene ID** | ***Glycine max* gene name** | ***Oryza sativa* gene location** | | | ***Oryza sativa* gene ID** | ***Oryza sativa* gene name** |
| **Chr** | **start** | **end** | | | **Chr** | **start** | **end** | | |
| Gm1 | 411125 | 413137 | Glyma.01G003900 | GmACS1 | Os5 | 5954550 | 5956246 | LOC_Os05g10780 | OsACS4 |

| | | | | | | | | | |
|---|---|---|---|---|---|---|---|---|---|
| Gm1 | 411125 | 413137 | Glyma.01G003900 | GmACS1 | Os1 | 5002117 | 5005727 | LOC_Os01g09700 | OsACS1 |
| Gm1 | 53027373 | 53030242 | Glyma.01G196100 | GmACS2 | Os3 | 29660661 | 29662927 | LOC_Os03g51740 | OsACS2 |
| Gm3 | 2719351 | 2719869 | Glyma.03G025100 | GmACS4 | Os4 | 29131809 | 29134682 | LOC_Os04g48850 | OsACS3 |
| Gm4 | 3941607 | 3943357 | Glyma.04G048700 | GmACS5 | Os3 | 29660661 | 29662927 | LOC_Os03g51740 | OsACS2 |
| Gm6 | 1526712 | 1529646 | Glyma.06G049900 | GmACS9 | Os3 | 29660661 | 29662927 | LOC_Os03g51740 | OsACS2 |
| Gm7 | 15313240 | 15315484 | Glyma.07G128000 | GmACS11 | Os1 | 5002117 | 5005727 | LOC_Os01g09700 | OsACS1 |
| Gm8 | 2411044 | 2413124 | Glyma.08G030100 | GmACS13 | Os1 | 5002117 | 5005727 | LOC_Os01g09700 | OsACS1 |
| Gm9 | 37521670 | 37525557 | Glyma.09G152700 | GmACS14 | Os4 | 29131809 | 29134682 | LOC_Os04g48850 | OsACS3 |
| Gm16 | 3050719 | 3054820 | Glyma.16G032200 | GmACS19 | Os6 | 1629716 | 1633629 | LOC_Os06g03990 | OsACS6 |
| Gm16 | 3050719 | 3054820 | Glyma.16G032200 | GmACS19 | Os4 | 29131809 | 29134682 | LOC_Os04g48850 | OsACS3 |
| Gm17 | 13554133 | 13556242 | Glyma.17G158100 | GmACS21 | Os3 | 29660661 | 29662927 | LOC_Os03g51740 | OsACS2 |

| ***Glycine max* gene location** | | | ***Glycine max* gene ID** | ***Glycine max* gene name** | ***Triticum aestivum* gene location** | | | ***Triticum aestivum* gene ID** | ***Triticum aestivum* gene name** |
|---|---|---|---|---|---|---|---|---|---|
| **Chr** | **start** | **end** | | | **Chr** | **start** | **end** | | |
| Gm1 | 411125 | 413137 | Glyma.01G003900 | GmACS1 | Os4 | 24261369 | 24267801 | TraesCS3A02G106600 | TaACS4 |
| Gm7 | 15313240 | 15315484 | Glyma.07G128000 | GmACS11 | Ta3A | 70848330 | 70850223 | TraesCS3A02G106600 | TaACS4 |
| Gm1 | 411125 | 413137 | Glyma.01G003900 | GmACS1 | Ta3A | 70848330 | 70850223 | TraesCS3B02G125200 | TaACS5 |

| | | | | | | | | | |
|---|---|---|---|---|---|---|---|---|---|
| Gm7 | 15313240 | 15315484 | Glyma.07G128000 | GmACS11 | Ta3B | 99500619 | 99502567 | TraesCS3B02G125200 | TaACS5 |
| Gm1 | 411125 | 413137 | Glyma.01G003900 | GmACS1 | Ta3B | 99500619 | 99502567 | TraesCS3D02G108700 | TaACS6 |
| Gm7 | 15313240 | 15315484 | Glyma.07G128000 | GmACS11 | Ta3D | 61802605 | 61804484 | TraesCS3D02G108700 | TaACS6 |
| Gm11 | 3379030 | 3381262 | Glyma.11G045600 | GmACS18 | Ta3D | 61802605 | 61804484 | TraesCS4A02G256500 | TaACS7 |
| Gm5 | 28858965 | 28861063 | Glyma.05G108900 | GmACS6 | Ta4A | 568192758 | 568195091 | TraesCS4A02G256500 | TaACS7 |
| Gm11 | 3379030 | 3381262 | Glyma.11G045600 | GmACS18 | Ta4A | 568192758 | 568195091 | TraesCS4B02G058200 | TaACS9 |
| Gm11 | 3379030 | 3381262 | Glyma.11G045600 | GmACS18 | Ta4B | 48991437 | 48993796 | TraesCS4D02G058200 | TaACS10 |
| Gm1 | 411125 | 413137 | Glyma.01G003900 | GmACS1 | Ta4D | 34088249 | 34091236 | TraesCS3A02G106600 | TaACS4 |
| ***Arabidopsis thaliana* gene location** | | | ***Arabidopsis thaliana* gene ID** | ***Arabidopsis thaliana* gene name** | ***Arabidopsis thaliana* gene location** | | | ***Arabidopsis thaliana* gene ID** | ***Arabidopsis thaliana* gene name** |
| **Chr** | **start** | **end** | | | **Chr** | **start** | **end** | | |
| At1 | 175781 | 178400 | AT1G01480 | AtACS1 | At3 | 22763494 | 22765730 | AT3G61510 | AtACS4 |
| At2 | 9717790 | 9719472 | AT2G22810 | AtACS2 | At5 | 26330745 | 26332812 | AT5G65800 | AtACS9 |
| At2 | 9717790 | 9719472 | AT2G22810 | AtACS2 | At4 | 17752083 | 17754157 | AT4G37770 | AtACS8 |
| At2 | 9717790 | 9719472 | AT2G22810 | AtACS2 | At3 | 18434469 | 18436141 | AT3G49700 | AtACS3 |
| At3 | 18434469 | 18436141 | AT3G49700 | AtACS3 | At5 | 26330745 | 26332812 | AT5G65800 | AtACS9 |
| At3 | 18434469 | 18436141 | AT3G49700 | AtACS3 | At4 | 17752083 | 17754157 | AT4G37770 | AtACS8 |
| At3 | 22763494 | 22765730 | AT3G61510 | AtACS4 | At4 | 6864096 | 6866116 | AT4G11280 | AtACS6 |

| | | | | | | | | | |
|---|---|---|---|---|---|---|---|---|---|
| At4 | 17752083 | 17754157 | AT4G37770 | AtACS8 | At5 | 26330745 | 26332812 | AT5G65800 | AtACS9 |
| ***Medicago truncatula* location** | | | ***Medicago truncatula* gene ID** | ***Medicago truncatula* gene name** | ***Medicago truncatula* gene location** | | | ***Medicago truncatula* gene ID** | ***Medicago truncatula* gene name** |
| **Chr** | **start** | **end** | | | **Chr** | **start** | **end** | | |
| Mt3 | 47826473 | 47828998 | Medtr3g103550 | MtACS2 | Mt5 | 5133747 | 5135930 | Medtr5g015020 | MtACS5 |
| Mt3 | 47826473 | 47828998 | Medtr3g103550 | MtACS2 | Mt4 | 40206877 | 40208967 | Medtr4g097540 | MtACS3 |
| Mt4 | 40206877 | 40208967 | Medtr4g097540 | MtACS3 | Mt5 | 5133747 | 5135930 | Medtr5g015020 | MtACS5 |
| Mt6 | 34566027 | 34568470 | Medtr6g091760 | MtACS6 | Mt8 | 42788673 | 42790665 | Medtr8g101750 | MtACS9 |
| Mt8 | 41419292 | 41422107 | Medtr8g098930 | MtACS8 | Mt8 | 10966038 | 10969861 | Medtr8g028600 | MtACS10 |
| Mt8 | 41419292 | 41422107 | Medtr8g098930 | MtACS8 | Mt7 | 29963155 | 29966691 | Medtr7g079080 | MtACS7 |
| Mt8 | 10966038 | 10969861 | Medtr8g028600 | MtACS10 | Mt7 | 29963155 | 29966691 | Medtr7g079080 | MtACS7 |
| ***Medicago truncatula* gene location** | | | ***Medicago truncatula* gene ID** | ***Medicago truncatula* gene name** | ***Oryza sativa* gene location** | | | ***Oryza sativa* gene ID** | ***Oryza sativa* gene name** |
| **Chr** | **start** | **end** | | | **Chr** | **start** | **end** | | |
| Mt4 | 40206877 | 40208967 | Medtr4g097540 | MtACS3 | Os3 | 29660661 | 29662927 | LOC_Os04g48850 | OsACS2 |
| ***Oryza sativa* gene location** | | | ***Oryza sativa* gene ID** | ***Oryza sativa* gene name** | ***Oryza sativa* gene location** | | | ***Oryza sativa* gene ID** | ***Oryza sativa* gene name** |
| **Chr** | **start** | **end** | | | **Chr** | **start** | **end** | | |
| Os5 | 5954550 | 5956246 | LOC_Os05g10780 | OsACS4 | Os1 | 5002117 | 5005727 | LOC_Os01g09700 | OsACS1 |

| *Triticum aestivum* gene location | | | *Triticum aestivum* gene ID | *Triticum aestivum* gene name | *Triticum aestivum* gene location | | | *Triticum aestivum* gene ID | *Triticum aestivum* gene name |
|---|---|---|---|---|---|---|---|---|---|
| **Chr** | **start** | **end** | | | **Chr** | **start** | **end** | | |
| Ta2A | 650243368 | 650246266 | TraesCS2A02G396400 | TaACS1 | Ta2B | 592801835 | 592804641 | TraesCS2B02G414800 | TaACS2 |
| Ta2A | 650243368 | 650246266 | TraesCS2A02G396400 | TaACS1 | Ta2D | 505814183 | 505817055 | TraesCS2D02G394200 | TaACS3 |
| Ta2B | 592801835 | 592804641 | TraesCS2B02G414800 | TaACS2 | Ta2D | 505814183 | 505817055 | TraesCS2D02G394200 | TaACS3 |
| Ta3A | 70848330 | 70850223 | TraesCS3A02G106600 | TaACS4 | Ta3B | 99500619 | 99502567 | TraesCS3B02G125200 | TaACS5 |
| Ta3A | 70848330 | 70850223 | TTraesCS3A02G106600 | TaACS4 | Ta3D | 61802605 | 61804484 | TraesCS3D02G108700 | TaACS6 |
| Ta3B | 99500619 | 99502567 | TraesCS3B02G125200 | TaACS5 | Ta3D | 61802605 | 61804484 | TraesCS3D02G108700 | TaACS6 |
| Ta4A | 568192758 | 568195091 | TraesCS4A02G256500 | TaACS7 | Ta4B | 48991437 | 48993796 | TraesCS4B02G058200 | TaACS9 |
| Ta4A | 568192758 | 568195091 | TraesCS4A02G256500 | TaACS7 | Ta4D | 34088249 | 34091236 | TraesCS4D02G058200 | TaACS10 |
| Ta4A | 693274378 | 693278701 | TraesCS4A02G422900 | TaACS8 | Ta7A | 33361446 | 33365622 | TraesCS7A02G066400 | TaACS11 |
| Ta4A | 693274378 | 693278701 | TraesCS4A02G422900 | TaACS8 | Ta7D | 33045597 | 33051044 | TraesCS7D02G060600 | TaACS12 |
| Ta4B | 48991437 | 48993796 | TraesCS4B02G058200 | TaACS9 | Ta4D | 34088249 | 34091236 | TraesCS4D02G058200 | TaACS10 |
| Ta7A | 33361446 | 33365622 | TraesCS7A02G066400 | TaACS11 | Ta7D | 33045597 | 33051044 | TraesCS7D02G060600 | TaACS12 |
| ***Triticum aestivum* gene location** | | | ***Triticum aestivum* gene ID** | ***Triticum aestivum* gene name** | ***Arabidopsis thaliana* gene location** | | | ***Arabidopsis thaliana* gene ID** | ***Arabidopsis thaliana* gene name** |
| **Chr** | **start** | **end** | | | **Chr** | **start** | **end** | | |
| Ta2A | 650243368 | 650246266 | TraesCS2A02G396400 | TaACS1 | At4 | 6864096 | 6866116 | AT4G11280 | AtACS6 |

| | | | | | | | | | |
|---|---|---|---|---|---|---|---|---|---|
| Ta2B | 592801835 | 592804641 | TraesCS2B02G414800 | TaACS2 | At4 | 6864096 | 6866116 | AT4G11280 | AtACS6 |
| Ta2D | 505814183 | 505817055 | TraesCS2D02G394200 | TaACS3 | At4 | 6864096 | 6866116 | AT4G11280 | AtACS6 |
| Ta3A | 70848330 | 70850223 | TTraesCS3A02G106600 | TaACS4 | At4 | 13275306 | 13277188 | AT4G26200 | AtACS7 |
| Ta3B | 99500619 | 99502567 | TraesCS3B02G125200 | TaACS5 | At4 | 13275306 | 13277188 | AT4G26200 | AtACS7 |
| Ta3D | 61802605 | 61804484 | TraesCS3D02G108700 | TaACS6 | At4 | 13275306 | 13277188 | AT4G26200 | AtACS7 |
| Ta4A | 568192758 | 568195091 | TraesCS4A02G256500 | TaACS7 | At2 | 9717790 | 9719472 | AT2G22810 | AtACS2 |
| Ta4B | 48991437 | 48993796 | TraesCS4B02G058200 | TaACS9 | At2 | 9717790 | 9719472 | AT2G22810 | AtACS2 |
| Ta4D | 34088249 | 34091236 | TraesCS4D02G058200 | TaACS10 | At2 | 9717790 | 9719472 | AT2G22810 | AtACS2 |

| ***Triticum aestivum* gene location** | | | ***Triticum aestivum* gene ID** | ***Triticum aestivum* name** | ***Medicago truncatula* gene location** | | | ***Medicago truncatula* gene ID** | ***Medicago truncatula* gene name** |
|---|---|---|---|---|---|---|---|---|---|
| **Chr** | **start** | **end** | | | **Chr** | **start** | **end** | | |
| Ta4A | 568192758 | 568195091 | TraesCS4A02G256500 | TaACS7 | Mt3 | 47826473 | 47828998 | Medtr3g103550 | MtACS2 |
| Ta4B | 48991437 | 48993796 | TraesCS4B02G058200 | TaACS9 | Mt3 | 47826473 | 47828998 | Medtr3g103550 | MtACS2 |
| Ta4D | 34088249 | 34091236 | TraesCS4D02G058200 | TaACS10 | Mt3 | 47826473 | 47828998 | Medtr3g103550 | MtACS2 |
